# Imaging lung regeneration by light sheet microscopy

**DOI:** 10.1007/s00418-020-01903-8

**Published:** 2020-07-19

**Authors:** Isabelle Salwig, Birgit Spitznagel, Marion Wiesnet, Thomas Braun

**Affiliations:** 1 Department of Cardiac Development and Remodeling, Max-Planck-Institute for Heart and Lung Research, Ludwigstr. 43, 61231 Bad Nauheim, Germany; 2 Member of the German Center for Lung Research (DZL), Giessen, Germany; 3 Member of the Cardio-Pulmonary Institute (CPI), Bad Nauheim, Frankfurt, Giessen, Germany

**Keywords:** Lung regeneration, Club cells, Light sheet microscopy, Optical clearing, Stem cell niches, Regenerative foci

## Abstract

Optical clearing combined with deep imaging of large biological specimen allows organ-wide visualization of cells in three dimensions (3D) to explore regenerative processes in a spatial context. Here, we investigate the dynamics of airway regeneration following toxin-mediated epithelial injury in cleared whole lung preparations by light sheet microscopy. We use a recently developed knock-in mouse strain labeling bronchiolar Club cells (*Scgb1a1*-mCherry) to define an optimal clearing procedure that efficiently preserves genetically encoded fluorophores. Dehydration in pH-adjusted tert-butanol followed by clearing in ethyl cinnamate maintained maximum mCherry fluorescence while preventing unfavorable background fluorescence. We apply this technique to depict the course of bronchiolar epithelial renewal from an acute injury phase to early and late recovery stages. 3D reconstructions of whole lungs demonstrate near-complete loss of secretory Club cells throughout the entire respiratory tract 3 days post naphthalene (dpn). Multiple foci of regenerating Club cells emerge at 7 dpn, predominantly at airway bifurcations and in distal terminal bronchioles—anatomical regions assumed to harbor distinct stem/progenitor cells subsets. At 21 dpn, clusters of newly formed Club cells have largely expanded, although the bronchiolar epithelial lining continues to regenerate. This study identifies regional stem cell niches as starting points for epithelial recovery, underscores the enormous regenerative capacity of the respiratory epithelium and demonstrates the power of whole lung 3D imaging for evaluating the extent of pulmonary damage and subsequent repair processes.

**Electronic supplementary material:**

The online version of this article (10.1007/s00418-020-01903-8) contains supplementary material, which is available to authorized users.

## Introduction

With every breath, lungs are exposed to potentially harmful air-borne pollutants and pathogens that may damage the airways. To cope with repetitive challenges and to rapidly replace lost or impaired cells, the pulmonary epithelium requires a high regenerative potential. Since its three-dimensional organization and cellular composition changes along a proximal-to-distal axis, efficient repair of the complex respiratory epithelium relies on the joint action of different regional stem/progenitor cell subsets. Proximal conducting airways are lined by a pseudostratified columnar epithelium mainly composed of secretory, ciliated, basal and neuroendocrine cells. The pseudostratified tracheobronchial epithelium decreases in height as airways undergo successive divisions, transitioning into a simple columnar epithelium. In distal conducting airways, Club cells predominate over ciliated cells and neuroendocrine cells are found as local innervated clusters, termed neuroepithelial bodies (NEBs), which tend to concentrate near airway bifurcations. At the bronchioalveolar duct junction (BADJ), the airway epithelium changes into a squamous alveolar epithelium composed of type 1 (AT1) and type 2 pneumocytes (AT2). Along this proximal-to-distal axis, multiple epithelial stem/progenitor cell types coexist that cooperatively contribute to epithelial repair, including basal cells in the trachea and proximal conducting airways (Rock et al. 2009), secretory Club cells in bronchi and bronchioles (Rawlins et al. 2009) and AT2 cells in the distal alveolar epithelium (Barkauskas et al. 2013). In addition to major epithelial cell populations that exert regular homeostatic functions but also serve as a source for cellular renewal, diverse niche-associated cell subsets with differing lineage potencies have been identified, which primarily become activated in response to regenerative cues. Such cell subsets include e.g. pollutant-resistant variant Club cells (vClub) at airway bifurcations (Reynolds et al. 2000), bronchioalveolar stem cells (BASCs) at BADJs (Kim et al. 2005; Salwig et al. 2019) and distal airway stem cells (DASCs)/lineage-negative progenitors (LNEPs) which serve as emergency backup in response to viral infection (Vaughan et al. 2015; Zuo et al. 2015).

To gain insights into regenerative processes not only at a cellular level, but also at an organ-wide scale, it is necessary to visualize the dynamics of distinct cell populations during lung regeneration at a three-dimensional (3D) spatial resolution. Acquisition of 3D data is of great importance, in particular for highly specialized organs with a complex architecture and varying regional (micro) environments. Only 3D imaging allows reliable detection of local, eventually rare events in large volumes and a better understanding of topological cellular interactions.

Clearing techniques that turn opaque tissues into highly transparent samples are a pre-requisite for deep imaging of large biological specimen. In recent years, a multitude of optical clearing procedures have been developed, which can be classified into organic solvent- and aqueous-based techniques (Richardson and Lichtman 2015). One major advantage of solvent-based clearing is the high degree of tissue transparency that can be achieved in short experimental periods (days), albeit the toxicity of many solvents, tissue-shrinkage due to dehydration and quenching of fluorescent protein emission limits its usefulness. Aqueous-based clearing approaches may preserve fluorophores more efficiently and prevent tissue-shrinkage, but are often limited to smaller specimens and require longer incubation times to achieve comparable transparency levels (weeks). Importantly, recent technical advances such as identification of non-toxic solvents with high refractive indices (≥ 1.5) and improved fluorescence preservation due to optimized dehydration procedures have overcome some of the main limitations of solvent-based clearing (Klingberg et al. 2017; Masselink et al. 2019).

In this study, we investigated the spatio-temporal pattern of airway regeneration in whole lung preparations by light sheet microscopy. We identified an optimized solvent-based clearing method that efficiently preserved fluorescence in *Scgb1a1*-mCherry animals, which enabled us to monitor Club cell depletion and recovery following experimental injury in a 3D context.

## Materials and methods

### Animal husbandry and naphthalene exposure

*Scgb1a1*-mCherry (CCSP^−2A mCherry−2A tTA C^) animals have been described previously (Salwig et al. 2019). Animals were housed in individual ventilated caging (IVC) systems on a 12‐h‐based light/dark cycle with food and water provided *ad libitum*. All animal experiments were performed in accordance with the Guide for the Care and Use of Laboratory Animals published by the US National Institutes of Health (NIH Publication No. 85‐23, revised 1996) and were approved by the local authorities. To induce depletion of bronchiolar Club cells, naphthalene (84679, Sigma) was dissolved in Miglyol^®^ 812 (3274, Caelo) and injected intraperitoneally at a dose of 200 mg naphthalene per kg body weight.

### Tissue isolation and optical clearing

Following *trans*‐cardial perfusion with PBS, lungs were cannulated via the trachea, inflated by instillation of formaldehyde solution (1% in PBS; 252549, Sigma), and fixed in situ for 5 min at constant pressure (infusion height 20 cm). After ligation of the trachea, lungs were dissected from the thoracic cavity and incubated for 2 h in fixative on ice. To stop fixation, ligature was removed and lungs were washed in PBS (> 1 h), followed by dehydration in increasing concentrations of different pH-adjusted alcohols (50–70–100–100%, 2 h each). To identify an optimal clearing procedure that provides the best signal-to-noise ratio (maximum fluorescence preservation but minimal background fluorescence), the following alcohols were compared for dehydration: ethanol (9065, Roth), 1-butanol (7171, Roth), 1-propanol (279544, Sigma) and tert-butanol (360538, Sigma). Alcohols were diluted in alkaline PBS (pH > 10, adjusted with NaOH) to achieve a final pH > 9. Lungs were incubated in increasing alcohol series at room temperature under gentle agitation, protected from light. After the last dehydration step, lungs were transferred into ethyl cinnamate (112372, Sigma) and immersed for > 1 h prior to imaging.

### Light sheet imaging and 3D reconstruction

Image acquisition of whole lung preparations was performed using an Ultramicroscope II (LaVision BioTec, Germany) equipped with an Olympus MVX-10 Zoom Body with 2 × objective (1.26 ×–12.6 × ), a bi-directional triple light sheet module and an Andor Zyla 4.2 Plus sCMOS camera (2048 × 2048 pixel, 6.5 µm × 6.5 µm pixel size). For detection of mCherry fluorescence, a 561 nm laser line and a 620/60 emission filter were used. Image acquisition was performed using the LaVision BioTec ImSpector software (version 5.1.328) while 3D reconstructions and export of movies were carried out using the Imaris software (version × 64 9.5.1.).

## Results and discussion

**Fig. 1:**
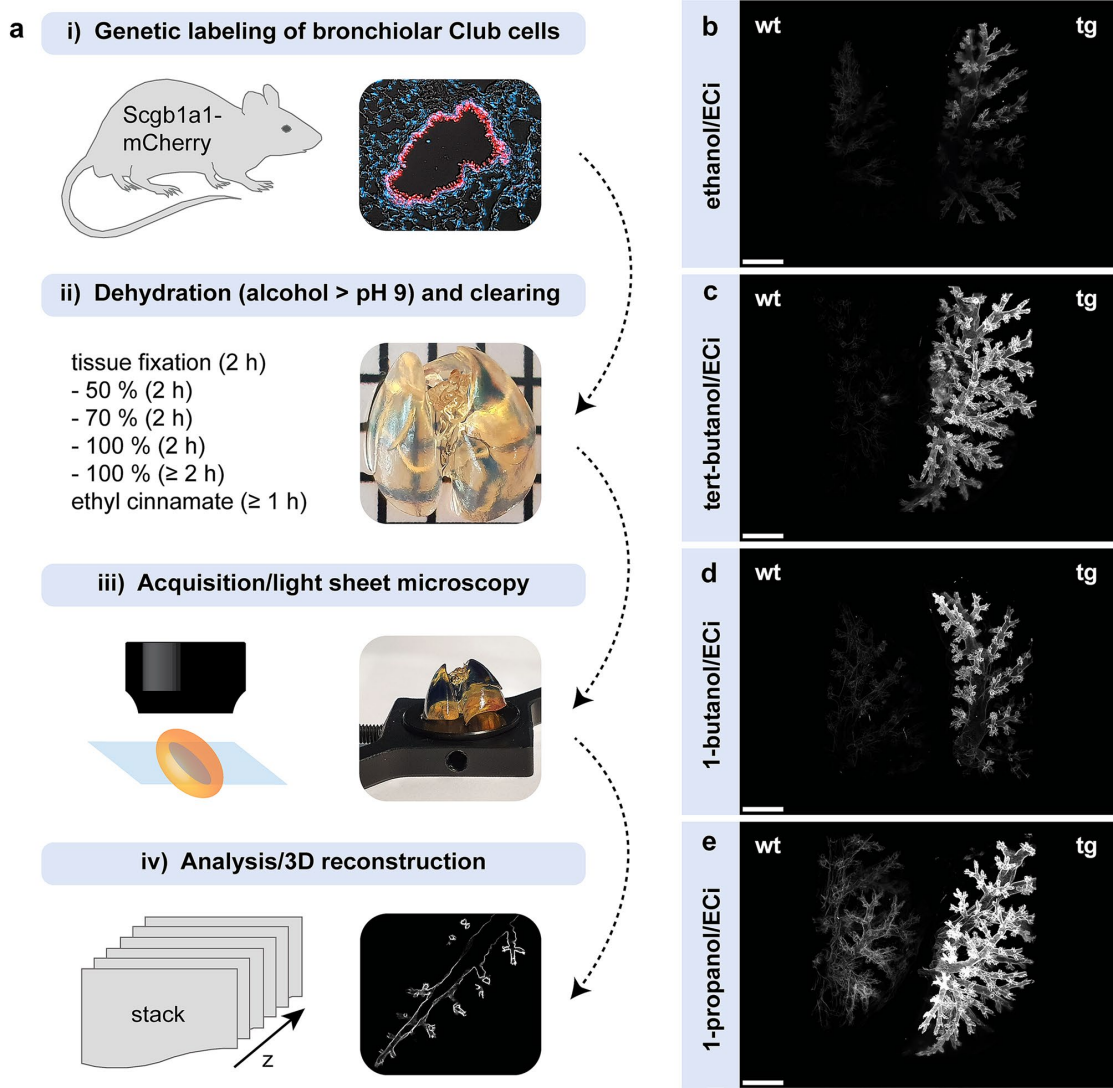
Comparison of fluorescence preservation in lungs of Club-cell specific reporter mice. **a** Schematic representation of the experimental procedure. To achieve tissue transparency, lungs were dehydrated using different pH-adjusted alcohol series and cleared in the organic solvent ethyl cinnamate (ECi). **b–e** 3D reconstructions of lung lobes after dehydration/clearing in ethanol/ECi (**b**), tert-butanol/ECi (**c**), 1-butanol/ECi (**d**) and 1-propanol/ECi (**e**). To evaluate the degree of auto- vs. epi-fluorescence, wildtype (wt) and transgenic (tg = *Scgb1a1*-mCherry) lung lobes were recorded simultaneously by light sheet microscopy. Scale bar: 500 µm

We recently established mouse models enabling selective genetic manipulation of dual-marker expressing BASCs based on intein-mediated split-effector reconstitution (Salwig et al. 2019). In addition to corresponding split-effector halves, C-terminal knock-in strains contain a fluorescent reporter gene integrated into the endogenous *Scgb1a1* locus, allowing direct visualization of Club cells throughout the airway tract (“*Scgb1a1*-mCherry”). 2D fluorescence microscopy of lung tissue sections demonstrates robust mCherry expression restricted to dome-shaped secretory cells in the bronchiolar epithelium (Fig. 1a). For 3D imaging of Club cells in whole lung preparations, we had to achieve maximum tissue transparency while optimally preserving mCherry fluorescence. The strong pH sensitivity of fluorescent proteins, particularly in an acidic milieu (Elsliger et al. 1999; Kneen et al. 1998), is a major reason for the dramatic loss of fluorescence frequently observed during optical clearing. To prevent fluorophore quenching, we adopted a clearing procedure based on dehydration in alcohol series adjusted to an alkaline pH, followed by refractive index matching using the non-toxic compound ethyl cinnamate (ECi) which had been demonstrated to yield high degrees of tissue transparency (Klingberg et al. 2017). In addition, we compared classical ethanol/ECi clearing with dehydration in other alcohols that previously showed improved preservation of fluorescence (Masselink et al. 2019). Following fixation, lungs of *Scgb1a1*-mCherry mice were dehydrated in increasing concentrations of pH-adjusted (pH > 9) ethanol, tert-butanol, 1-butanol or 1-propanol and cleared by immersion in ECi, resulting in highly transparent specimens (Fig. 1a). Subsequently, lungs were imaged by light sheet microscopy applying bi-directional sheet excitation for consistent illumination. Acquired image stacks were then processed to reconstruct 3D views of entire lung lobes. For direct comparison of remaining auto- vs. preserved epi-fluorescence, cleared wildtype (wt) and transgenic (tg) *Scgb1a1*-mCherry lung lobes were fixed side-by-side on the sample holder and recorded simultaneously (Fig. 1b–e). Despite alkaline refractive index matching, classical ethanol/ECi clearing resulted in substantial quenching of mCherry fluorescence, accompanied by a loss of structural and morphological details (Fig. 1b). In contrast, tert-butanol/ECi clearing efficiently preserved mCherry fluorescence without substantial auto-fluorescence, allowing a clear delineation of the bronchiolar tubing throughout the entire reconstructed lung lobe (Fig. 1c). Partial quenching of the fluorophore was also observed following dehydration in 1-butanol (Fig. 1d), although to a lower extent than standard ethanol/ECi clearing. Maximum fluorescence preservation was achieved using 1-propanol/ECi clearing, but this approach concomitantly caused unfavorable levels of background fluorescence (Fig. 1e). We therefore selected tert-butanol/ECi clearing for subsequent analyses, which showed the best signal-to-noise ratio (Fig. 1b–e).

**Fig. 2:**
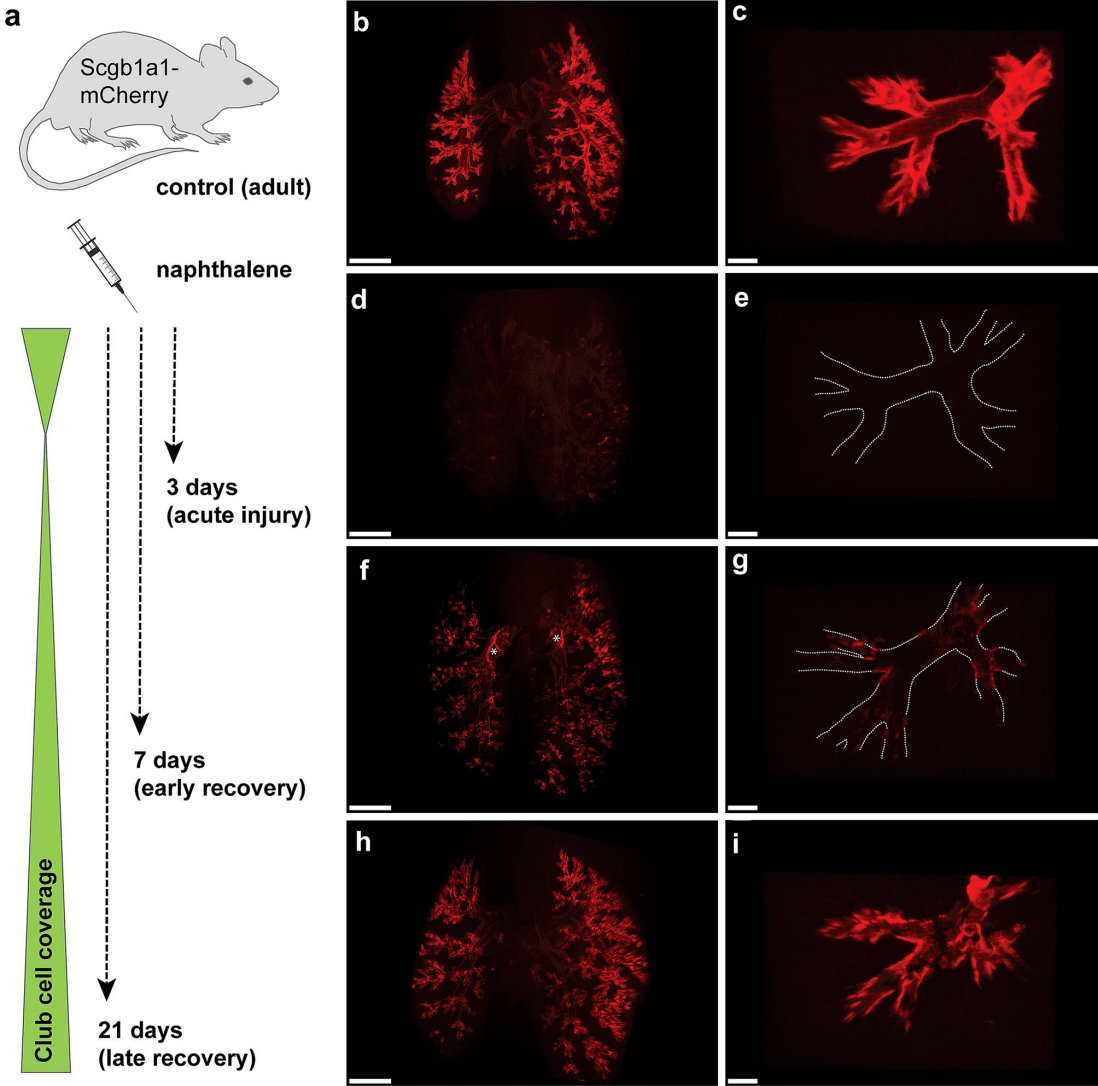
Visualization of Club cell depletion and recovery in whole lungs following experimental injury. **a** Schematic representation of the experimental procedure. To induce bronchiolar damage, *Scgb1a1*-mCherry animals were challenged by single intraperitoneal injection of naphthalene (200 mg/kg). Lungs were harvested at different days post naphthalene (dpn). **b–i** 3D reconstructions of whole lung preparations (**b, d, f, h**; scale bar: 2 mm) and magnified areas (**c, e, g, i**; scale bar: 200 µm) isolated from control (**b-c**) and injured *Scgb1a1*-mCherry animals at 3 dpn (**d–e**), 7 dpn (**f–g**) and 21 dpn (**h–i**). Dotted lines depict bronchiolar boundaries, asterisks mark air inclusions with intrinsic background fluorescence

Next, we studied the dynamics of airway regeneration after experimental injury in whole lung preparations (Fig. 2). To induce bronchiolar damage, *Scgb1a1*-mCherry animals were treated with the organic compound naphthalene, a cytotoxicant that results in selective depletion of secretory Club cells. The strong susceptibility of Club cells to naphthalene is mainly due to the high expression of cytochrome P450 monooxygenases, in particular of CYP2F2, which metabolizes naphthalene to its toxic products (Fanucchi et al. 1997; Shultz et al. 1999; Buckpitt et al. 1995). Maximum loss of Club cells is typically observed within 48 h after treatment, followed by recovery of the bronchiolar epithelium, which is nearly completed after three weeks (Stripp et al. 1995). Lungs of challenged animals were harvested 3, 7 and 21 days post naphthalene (dpn), corresponding to an acute injury phase as well as to early and late recovery stages (Fig. 2a), cleared via tert-butanol/ECi and subjected to light sheet microscopy. In non-injured control lungs, mCherry^+^ Club cells homogenously covered the entire conducting tract, enabling the precise demarcation of the highly complex, branching architecture of the respiratory tree, spanning from proximal intralobar bronchi down to the tips of most distal terminal bronchioles (Fig. 2b, c). This picture changed dramatically at 3 dpn when virtually all secretory Club cells were destroyed, suggesting acute and wide-spread bronchiolar damage (Fig. 2d, e, Online Resource 1). Only few dispersed patches of mCherry^+^ cells were detected, presumably representing exfoliated (dying) Club cells rather than surviving clusters. Notably, we did not observe any difference in proximal–distal responsiveness, indicating that Club cells at all levels of airway generations are similarly affected by parenteral administration of naphthalene at the selected dose. Following extensive damage in the acute injury phase, at 7 dpn we observed initiation of epithelial recovery, with multiple foci of nascent Club cells emerging predominantly at airway bifurcations throughout the respiratory tree and in distal terminal bronchioles (Fig. 2f, g, Online Resource 2). Interestingly, previous studies identified different niche-associated, pollutant-resistant subsets of Scgb1a1-expressing cells as starting points for bronchiolar repair processes (Giangreco et al. 2002; Kim et al. 2005; Reynolds et al. 2000). vClub cells reside in close proximity to NEBs at bifurcations in more proximal conducting airways while BASCs localize at BADJs in distal terminal bronchioles. vClub cells as well as BASCs are characterized by low CYP2F2 expression levels (an important determinant for xenobiotic-resistance), thus survive toxin exposure and expand at the onset of epithelial repair processes, suggesting an essential role during airway regeneration (Reynolds et al. 2000; Salwig et al. 2019). Intriguingly, the pattern of regenerative foci observed in 3D reconstructions of whole lungs at 7 dpn closely matched the anatomical positions proposed to harbor bronchiolar stem/progenitor cell subsets (Fig. 2f, g, Online Resource 2). We therefore reason that newly formed Club cells at airway bifurcations and in distal terminal bronchioles mostly originate from vClub cells and BASCs, respectively. As epithelial repair proceeded, mCherry^+^ clusters extended from the well-defined regional niches, resulting in prominent coverage of airways with secretory Club cells at 21 dpn, although bronchiolar recovery was not fully completed at this stage (Fig. 2h, i, Online Resource 3). These results highlight the remarkable regenerative capacity of the respiratory epithelium that—despite extensive damage—is able to rapidly replenish the majority of lost Club cells within three weeks.

In conclusion, this work demonstrates that optical clearing based on alkaline dehydration in tert-butanol (pH > 9) and refractive index matching in non-toxic ECi is an excellent approach to generate highly transparent specimens for light sheet microscopy. The protocol requires little hands-on-time (< 1 day) and results in efficient preservation of genetically encoded fluorophores. The light sheet approach allows fast volumetric imaging of large biological specimens to identify cell subsets based on fluorescence reporter gene expression without the requirement of additional antibody-based staining. Cellular resolution can be achieved to certain degree but falls behind regular fluorescence microscopy on sections, which is a clear disadvantage when individual cells need to be visualized. In the present study detection of fluorescence primarily reflects small clusters of regenerating cells due to a trade-off between axial resolution and usable field of view (Power and Huisken 2017), which was sufficient to visualize lung regeneration after depletion of Club cells. Analysis of epithelial renewal in whole lungs unveiled critical features of airway regeneration, including (i) the extent of bronchiolar damage in the acute injury phase, (ii) proximal–distal responsiveness of secretory cells to toxin exposure, (iii) precise localization of emerging regenerative patches and (iv) the degree of Club cell coverage at different recovery stages. Altogether, 3D visualization highlights the enormous regenerative potential of the airway epithelium and indicates that epithelial recovery is orchestrated by distinct stem/progenitor cell subsets residing in regional niches along a proximal-to-distal axis of the respiratory tree.

## Electronic supplementary material

Below is the link to the electronic supplementary material.

Supplementary file1 Online Resource 1: Dramatic loss of secretory Club cells in response to toxin-exposure. The animated movies were created from 3D reconstructions of endogenous mCherry fluorescence in cleared whole lung preparations isolated from non-injured (ctrl.) and naphthalene-challenged *Scgb1a1*-mCherry animals at 3 dpn (acute injury phase). (MPEG 59928 kb)

Supplementary file2 Online Resource 2: Regenerating Club cells emerge as patches at distinct anatomical positions during the initial recovery phase. The animated movies were created from 3D reconstructions of endogenous mCherry fluorescence in cleared whole lung preparations isolated from non-injured (ctrl.) and naphthalene-challenged *Scgb1a1*-mCherry animals at 7 dpn (early recovery stage). (MPEG 59928 kb)

Supplementary file3 Online Resource 3: Enhanced Club cell coverage corresponds to advanced bronchiolar regeneration of the airway tract. The animated movies were created from 3D reconstructions of endogenous mCherry fluorescence in cleared whole lung preparations isolated from non-injured (ctrl.) and naphthalene-challenged *Scgb1a1*-mCherry animals at 21 dpn (late recovery stage). (MPEG 59928 kb)

## Data Availability

The data sets generated and/or analyzed during the current study are available from the corresponding author on reasonable request.
